# Application of BP Artificial Neural Network in Preparation of Ni–W Graded Coatings

**DOI:** 10.3390/ma14226781

**Published:** 2021-11-10

**Authors:** Pei Feng, Yuhua Shi, Peng Shang, Hanjun Wei, Tongtong Peng, Lisha Pang, Rongrong Feng, Wenyuan Zhang

**Affiliations:** 1 School of Equipment Management and Support, Engineering University of PAP, Xi’an 710078, China; 228fly@sina.com (Y.S.); victoriapeng@163.com (T.P.); plscau@foxmail.com (L.P.); alwaysrong@163.com (R.F.); 2 Science and Technology on Plasma Dynamics Lab, Air Force Engineering University, Xi’an 710038, China; imgblue1997@163.com; 3 School of Mechanical and Precision Instrument Engineering, Xi’an University of Technology, Xi’an 710048, China; 4 School of Mechanical Engineering, Xi’an Jiaotong University, Xi’an 710049, China; 5 Science and Technology on Thermostructural Composite Materials Laboratory, Northwestern Polytechnical University, Xi’an 710072, China; wei_hanjun@yeah.net

**Keywords:** Ni–W graded coatings, backward propagation (BP) neural network, pulse electrodeposition, high temperature oxidation

## Abstract

The internal stress difference between soft-ductile aluminum alloy substrate and hard-brittle Ni–W alloy coating will cause stress concentration, thus leading to the problem of poor bonding force. Herein, this work prepared the Ni–W graded coating on aluminum alloy matrix by the pulse electrodeposition method in order to solve the mechanical mismatch problem between substrate and coatings. More importantly, a backward propagation (BP) neural network was applied to efficiently optimize the pulse electrodeposition process of Ni–W graded coating. The SEM, EDS, XRD, Vickers hardness tester and Weighing scales are used to analyze the micromorphology, chemical element, phase composition, and micro hardness as well as oxidation weight increase, respectively. The results show that the optimal process conditions with BP neural network are as follows: the bath temperature is 30 °C, current density is 15 mA/cm^2^ and duty cycle is 0.3. The predicted value of the model agrees well with the experimental value curve, the relative error is minor. The maximum error is less than 3%, and the correlation coefficient is 0.9996. The Ni–W graded coating prepared by BP neural network shows good bonding with the substrate which has flat and smooth interface. The thickness of the coating is about 136 μm, which slows down the oxidation of the substrate and plays an effective role in protecting the substrate.

## 1. Introduction

**Figure 1:**
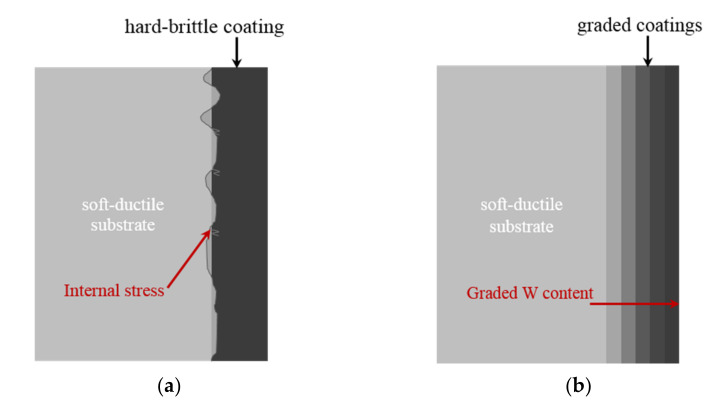
The bond between soft-ductile substrate and hard-brittle coating: (**a**) the internal stress of alloy coating; (**b**) superior bond of graded coating.

Aluminum alloy has been widely used in the mechanical manufacturing and aerospace fields due to its advantages of low density, superior electrical and thermal conductivity and optimal ductility [1,2,3]. However, certain disadvantages, such as low hardness and the fact that it is easily oxidized at high temperature, limit its service life and wide application [4]. Protective coating can be prepared on the surface of aluminum alloy to solve this problem. In this case, Ni–W coating has attracted extensive attention due to its excellent hardness, corrosion resistance and high temperature oxidation resistance as well as other properties [5,6,7,8,9]. In particular, pulse electrodeposition of Ni–W coatings shows the superiority of fine crystallization, smooth and bright and fast film formation rate, which could enhance the hardness, high temperature oxidation resistance of aluminum alloy substrates [10,11,12,13]. Nevertheless, prior research has shown that the mechanical differences between a soft-ductile substrate and hard-brittle coating could generate high mechanical mismatch between coatings and substrate, resulting in high-stress concentration and finally failure of the interface [14,15,16,17], as shown in Figure 1.

Ni–W graded coating with W gradient structure which can improve the bonding strength of coating and substrate [18], thermal shock resistance and fracture toughness [19]. In the preparation of Ni–W graded coatings, the process parameters can be determined by multiple tests which will cause an increase in cost, reduced efficiency as well as the polluting of the environment [20,21,22,23,24]. Therefore, Artificial Neural Network (ANN) can be introduced into the preparation of pulsed electrodeposition Ni–W graded coating. ANN technology can simulate some intelligent behaviors of the human brain and has the abilities of self-organization, self-learning, self-adaptation, high fault tolerance and highly nonlinear description. In conclusion, ANN is an effective method to deal with highly nonlinear and non-obvious functional relationship problems. It can model complex nonlinear processes efficiently and accurately [25,26].

Jiang et al. [25] applied a three-layer backward propagation (BP) model to predict the hardness of Ni-TiN nano coatings fabricated by pulse electrodeposition. Liu et al. [26] studied an ANN model to predict the HVOF of sprayed Cr_3_C_2_-25NiCr coating and to analyze the influence of operating parameters.

However, there are few studies on the relationships between the pulse electrodeposition and properties of Ni–W graded coating prepared by ANN. This paper established a network model of different process parameters to counter the influence of high-temperature oxidation performance using back-propagation neural network technology. The model was applied to predict and analyze performance on Ni–W coating. The technological parameters of pulsed electrodeposition Ni–W coating were optimized. Furthermore, the Ni–W graded coating was prepared by the optimized parameters. The microstructure, high-temperature oxidation resistance as well as micro-hardness of the coatings were observed and analyzed, respectively.

## 2. Materials and Methods

### 2.1. Materials

**Figure 2:**
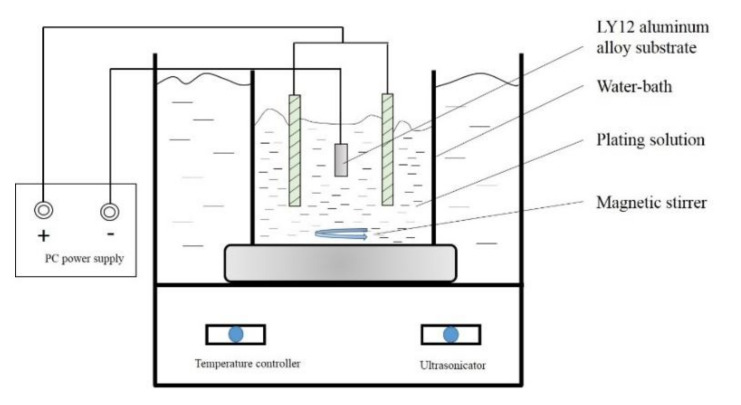
Schematic diagram of pulse electrodeposition battery.

Ni–W graded coatings were prepared by pulse electrodeposition technique. The purity of 99% nickel plate was used as the anode and LY12 aluminum alloy substrate as the cathode, with the size of 12 mm × 10 mm × 10 mm. The pulse electrodeposition parameters such as duty cycle, frequency, temperature and current density hold great influence on the properties of Ni–W coatings. The general pulse electrodeposition battery schematic diagram is shown in Figure 2.

### 2.2. Bath Composition and Plating Parameters

The chemical composition of bath and pulse electrodeposition parameters are listed in Table 1. The sample was immersed in 3.5 wt % NaCl solution at 25 °C, and the Ni–W graded coatings were subjected to corrosion test. The high temperature oxidation resistance of the coating was measured by cyclic oxidation weight gain method, the quality difference of the sample before and after heating was measured. The oxidation rate was the weight gain of the sample per unit area, and the unit was mg/cm^2^. The KSY muffle furnace is used in the experiment. The temperature of the electric furnace is automatically controlled by the temperature controller. Heat the resistance furnace to 400 °C, and put the prepared sample according to certain process parameters into the box resistance furnace to heat preservation. After cooling at room temperature, the samples were weighed on the Sartorius electronic balance (precision 0.01 mg). The mass of the samples after high-temperature oxidation was recorded. The difference between the initial mass and the initial mass was divided by the surface area of the sample, which was the oxidation weight gain of the sample, which was calculated by the Equation (1). The weight loss was measured by a sensitive electronic balance with an accuracy of 0.01 mg.

(1) $$\Delta M=\frac{M_{2}-M_{1}}{S}$$

where Δ*M* is the oxidation weight gain of the coating (mg/cm^2^), Δ*M*_1_ is the mass of the sample before high-temperature oxidation (g), Δ*M*_2_ is the mass of the sample after high-temperature oxidation (g), *S* is the surface area of the sample (cm^2^). Model inputs and symbols are shown in Appendix A, Table A1.

### 2.3. Composite Coating Characterization

Ni–W coating phase structures were analyzed by XRD-7000 from a Japan-Shimadzu X ray diffractometer using the Cu K alpha radiation to determine, with scanning speed of 4°/min, and scanning angle ranging from 20°–80°. Scanning electron microscope (SEM) and energy disperse spectroscopy (EDS) (JSM-6390A, Osaka, Japan) methods were also used to investigate the influence of pulse electrodeposition parameters on the microstructure and elements of Ni–W coatings. Abbreviation explanation are shown in Appendix A, Table A2.

## 3. Design of Test

As for the high temperature oxidation resistance of Ni–W coatings, the oxidation rate is mainly manifested as the oxidation weight gain of the coating surface. The smaller the oxidation weight gain of the plating layer, the better the oxidation resistance. The prediction of the BP neural network is generally analyzed by taking the maximum point. Therefore, in order to cooperate with the training and learning of BP neural network, Equation (2) is adopted to conduct reciprocal processing of the data.

*V* = 1/*m*_x_ (2)

Among them, m_x_ is the oxidative weight gain, and V is the reciprocal of the oxidative weight gain. The data processed by Equation (2) were shown in Table 2. In this paper, L9 (3^3^) orthogonal table is used for orthogonal experiments. The factors and level selection of orthogonal tests are shown in Table 3, and the test results are shown in Table 4.

## 4. BP Neural Network Structure

BP neural network is the most widely used and mature ANN that can deal with complex nonlinear systems rapidly. It is generally composed of three layers: the hidden layer, the input layer and the output layer. BP neural network also includes the forward propagation and the back propagation. Through the input layer, the experimental data are gradually propagated to the output layer of the hidden layer under the action of the excitation function. If the expected data are not obtained, it is transferred to back propagation by correcting the corresponding weights and thresholds several times, and finally the output value is within the allowable range of error.

### 4.1. Design of BP Neural Network

**Figure 3:**
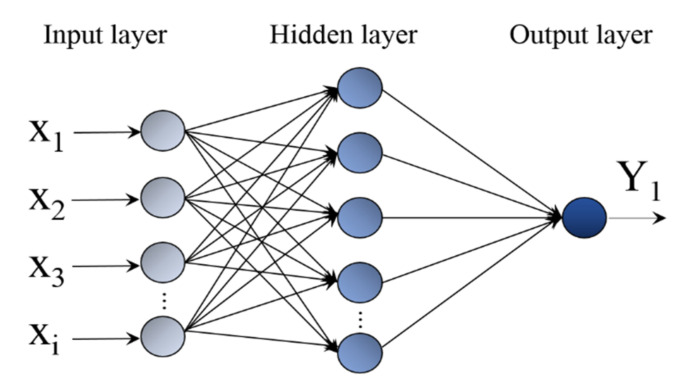
Schematic description of artificial neural network configuration.

There are many factors affecting the Ni–W coating, such as the bath temperature, PH, density, deposition time, duty ratio and so on. In this paper, the three factors of bath temperature, current density and duty cycle are set as the input sample, and the output sample is the reciprocal V of oxidation weight gain of the coating, as shown in Figure 3.

### 4.2. Network Training and Prediction

#### 4.2.1. Data Processing

After the input and output variables are determined, premnmx is used for normalization processing so that the results are between [0, 1]. After training and simulation, the data are subjected to anti-normalization processing.

#### 4.2.2. Network Training and Prediction

In this paper, the momentum BP algorithm with variable learning rate is adopted. The training function of this algorithm is Traingdx, and the hidden layer function adopts the differentiable hyperbolic tangent function. The hidden layer and the output layer adopted a linear function. During the training process, the maximum training times were selected to be 1000, and the learning rate was 0.05.

**Figure 4:**
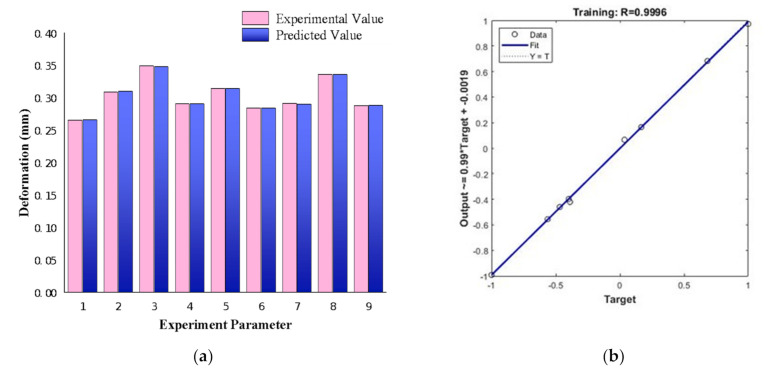
(**a**) Comparison of experimental data with predicted values; (**b**) Experimental values and curve fitting of predicted values.

It can be seen from Table 5 that the predicted value is quite consistent with the experimental value, and the relative error is small. This indicates that the model can well describe the mapping relationship between input and output. A relatively stable network was obtained, which could be used to predict the high-temperature oxidation resistance of Ni–W coating and provide an effective basis for the actual process, as shown in Figure 4a. The fitting effect of BP neural network is shown in Figure 4b, and its value is R = 0.9996.

### 4.3. Prediction of BP Neural Network

**Figure 5:**
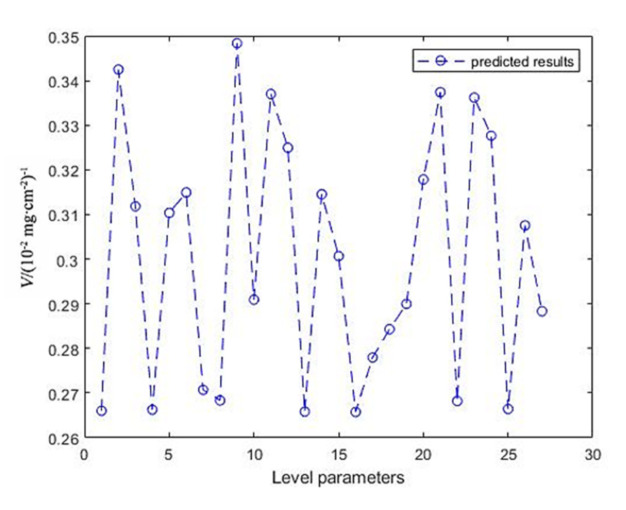
Prediction results of BP neural network.

It can be seen from Figure 5 that the BP neural network prediction curve is undulating, indicating that the combination at different technological levels has a significant impact on the rate of antioxidant weight gain of the coating. The 9th parameter combination at the highest point of BP neural network obtained the oxidation weight gain of 2.869 (10^−2^ mg/cm^2^) after treatment, which was not different from the actual measured value of 2.861 (10^−2^ mg/cm^2^). Therefore, the group 9th (T_1_C_3_D_3_), with the bath temperature of 30 °C, current density of 15 mA/cm^2^ and duty cycle of 0.3, can be determined as the best pulse electrodeposition parameter combination which can prepare the best oxidation resistance Ni–W coating. The Ni–W alloy coating and Ni–W gradient coating were prepared by using the best parameter combination simulated by the BP neural network.

## 5. Results and Discussion

### 5.1. The Cross-Sectional Morphology and EDS Spectra Analysis of the Prepared Ni–W Coatings

**Figure 6:**
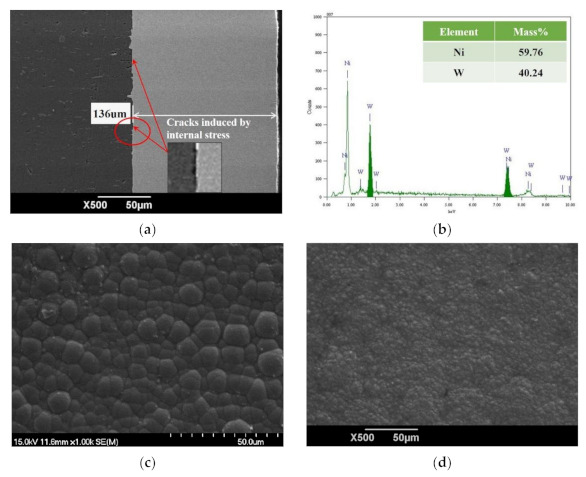
SEM images and EDS of Ni–W alloy coatings: (**a**) Section morphology; (**b**) EDS spectra; (**c**) Surface morphology before oxidation; (**d**) Surface morphology after oxidation at 400 °C/8 h in air.

The Ni–W alloy coating and further Ni–W graded coating were prepared by the optimal pulse electrodeposition parameters (T_1_C_3_D_3_), with the bath temperature of 30 °C, current density of 15 mA/cm^2^ and duty cycle of 0.3 simulated by BP neural network. The surface morphology of the Ni–W alloy coating was observed by scanning electron microscopy (SEM), as shown in Figure 6a. The light-colored area was Ni–W alloy coating, and the dark area was aluminum alloy matrix. Moreover, the distribution of the elements was uniform with the thickness of 136 μm. In addition, the obvious cracks, pores or other defects induced by high mechanical mismatch of soft-ductile substrate and hard-brittle coating are observed in Figure 6a. As shown in Figure 6b, the coatings consist of Ni element of 58.51% and W element of 41.49%, which further prove that the BP neural network successfully simulates the superior pulse electrodeposition parameters to prepare the Ni–W coating efficiently. Figure 6c,d show the surface morphology of Ni–W coatings before and after 400 °C heat treatment. The surface morphology of Ni–W coating shows the typical tightly packed cellular structure [27]. Apparently, Ni–W coating shows grain refinement after 400 °C heat treatment, which proves that the optimal parameters simulated by the BP neural network were effective in the experiments [28].

**Figure 7:**
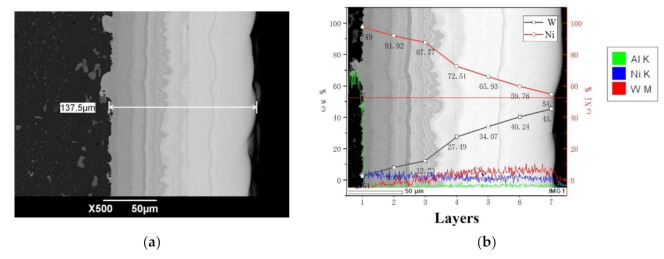
(**a**) The cross-section SEM micrographs; (**b**) The EDS of Ni–W graded coating.

Based on the BP neural network simulating the optimal pulse electrodeposition parameters, Ni–W graded coatings were further successfully prepared, as shown in Figure 7. The coating was divided into seven layers in total, and every layer was prepared using the optimal parameters simulated by BP neural network with different Na_2_WO_4_·2H_2_O addition, as shown in Table 1. Figure 7a shows the SEM image of Ni–W graded coating cross section morphology. It can be seen that Ni–W graded coatings were both closely bound to the matrix. The total thickness of the coating was about 137 m, the most left black area was aluminum alloy matrix, and the gradient ramp part was Ni–W graded coating (the brighter part of the coating, the higher the content of W, the lower the content of Ni) [29]. The boundary between the layers was tight and uniform without cracks. Figure 7b shows the line energy spectrum analysis which further proves the gradient W content in the coating. Compared with high mechanical mismatched Ni–W alloy coating, Ni–W graded coating shows better adhesion between coatings and matrix as well as different layers [30].

**Figure 8:**
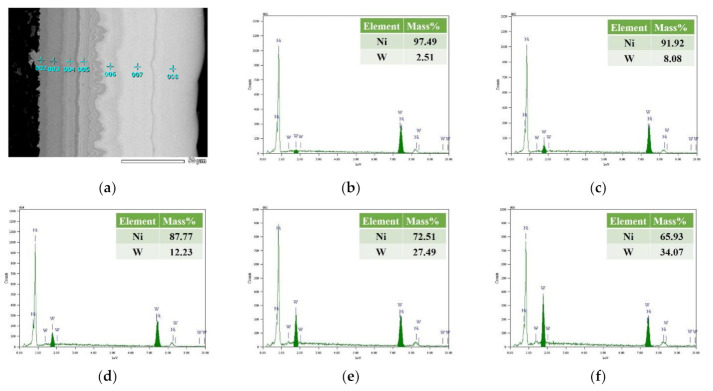

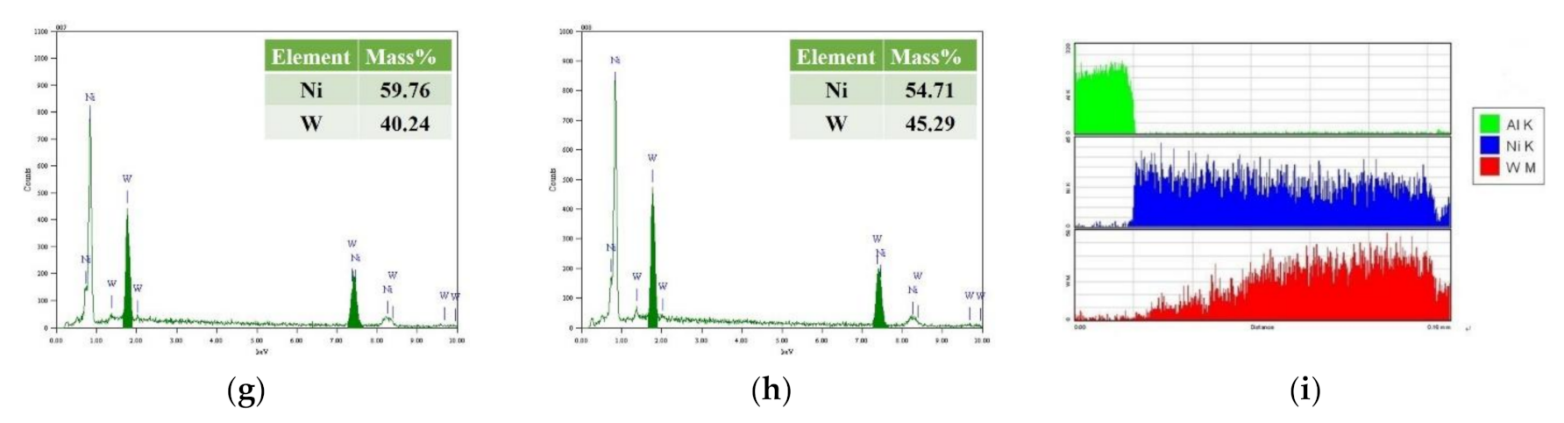
The EDS spectra of Ni–W graded coating: (**a**) the SEM of Ni–W graded coating, (**b**–**h**) the EDS spot spectra of different layers from first layer to seventh layer, (**i**) the EDS line spectra of Ni–W graded coating.

The EDS spectra of seven layers of Ni–W graded coatings are shown in Figure 8a–h. The content of W increased significantly from the 1st layer of 2.51% to the seventh layer of 45.29% among the Ni–W graded coating. The gradient W content not only affects the color of the coating, but also changes the micro-structure of Ni–W coating [5]. Figure 8i shows the gradient increase of W element trend and gradient decrease of Ni element trend in the prepared Ni–W graded coating. Several researchers demonstrate that the coating displays an amorphous state when the W% > 44% in the coatings [31]. Amorphous alloy coatings exhibit excellent physical and chemical properties due to their unique structural characteristics, such as excellent mechanical properties, wear and corrosion resistance, high resistivity, high activity and catalytic properties, and many special magnetic properties [10].

### 5.2. The XRD of Ni–W Graded Coatings

**Figure 9:**
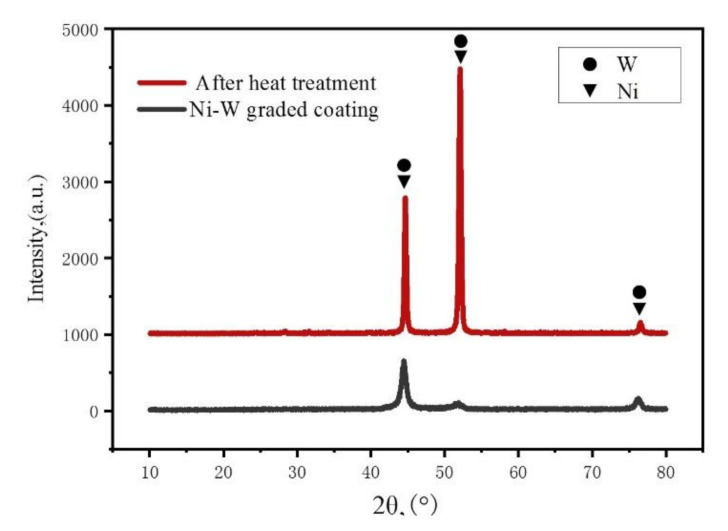
XRD of Ni–W alloy coating and Ni–W graded coating.

To further investigate the phase composition of Ni–W graded coating, XRD of the prepared samples before and after heat treatment are shown in Figure 9. Three diffraction peaks observed in Ni–W graded coating appear at diffraction angles of 2θ_1_ = 43.86°, 2θ_2_ = 51.40°, 2θ_3_ = 75.40°. As we know, the main diffraction peaks of pure Ni are located at 2θ_1_ = 44.62°, 2θ_2_ = 51.94°, 2θ_3_ = 76.14°, corresponding to the crystal planes of (1 1 1), (2 0 0) and (2 2 0). It can be seen that peaks of the Ni–W graded coating are similar to that of pure Ni. The atom of tungsten is larger than that of nickel [32]. Therefore, the solution of tungsten causes the expansion of the Ni lattice, which leads to the shift of the Ni diffraction peak, further indicating that the Ni–W coating is the solid solution structure [33,34]. Obviously, the content of W in the coating significantly influences the structure and the properties of the coating. The gentle diffraction peaks of the Ni–W graded coating further prove that it has an amorphous structure [35]. Apparently, when the samples were processed by heat treatment of 400 °C, 8 h, the three diffraction peaks become sharper, indicating that Ni–W graded coating becomes crystallized after heat treatment [26].

### 5.3. The High Temperature Oxidation Resistance of Ni–W Graded Coatings

**Figure 10:**
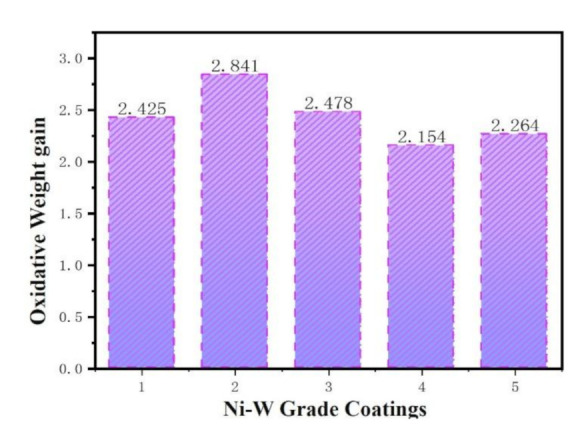
Experimental results verified the oxidation weight gain of BP optimized parameters.

To further verify the optimal pulse electrodeposition simulated by the BP neural network, we also weighed the samples after heat treatment for 400 °C, 8 h, as shown in Figure 10. The oxidative weight gains were calculated by Equation (1) and the average weight gain is 2.4324 mg/cm^2^, which is lower than original samples of average weight gain of 3.3194 mg/cm^2^, indicating the better high temperature oxidation resistance optimized by BP neural network. The reason for this enhancement is that the graded coating results in a denser coating with increased bonding and thus improved high temperature resistance to oxidation compared to a normal alloy coating with a relaxed bond.

### 5.4. The Micro-Hardness of Ni–W Graded Coatings

**Figure 11:**
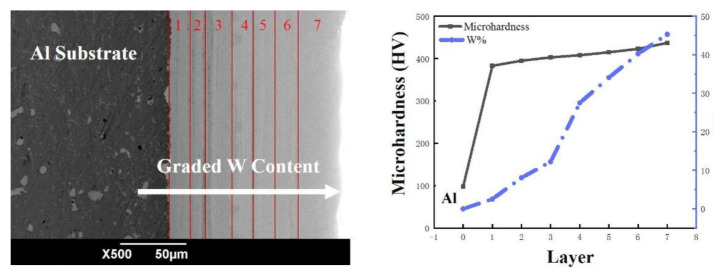
The micro-hardness changes with W content in Ni–W graded coating.

Figure 11 shows the Vickers hardness of the obtained Ni–W graded coatings, and the micro-hardness changes with W content in Ni–W graded coating. As presented in Figure 10, the pure Al alloy without Ni–W coating shows 98.7 HV, and it soars to 383.3 HV with the 1st layer Ni–W graded coating. As the W content increases from 2.51% to 45.29%, the hardness of the coating enhances from 383.3 HV to 437.5 HV, indicating that the hard tungsten contributes to the hardness of coatings [36,37]. The study shows that when the content of W in the coating increases, the degree of lattice distortion of the alloy increases, and the resistance of dislocation movement also increases, which leads to the increase of the micro-hardness of the alloy [38,39].

## 6. Conclusions

This work successfully prepared the Ni–W coatings by pulse electrodeposition. A BP neural network was used to optimize the parameters of pulse electrodeposition with high temperature oxidation resistance as index. The optimization results show that the ninth group 9th (T_1_C_3_D_3_), with the bath temperature of 30 °C, current density of 15 mA/cm^2^ and duty cycle of 0.3, were simulated as the optimal pulse electrodeposition parameter. The predicted value of the model agrees well with the experimental value curve, and the relative error is minor. The maximum error is less than 3%, and the correlation coefficient is 0.9996. According to the SEM and EDS test, Ni–W graded coatings including seven layers of gradient W content were successfully prepared, which effectively decreases the internal stress difference between soft-ductile substrate and hard-brittle coating. Furthermore, the weight gain test proves that BP neural networks can truly optimize the pulse electrodeposition parameters to prepare better high temperature oxidation resistance samples. Consequently, using BP neural networks to select the optimal preparation parameters and apply the optimal parameters to scientific research can greatly reduce cost and improve efficiency.

## Figures and Tables

**Table 1:** Chemical composition and plating conditions.

Chemical Composition	Content (g/L)	Plating Conditions	Parameters
NiSO_4_·7H_2_O	30 g/L	Pulse current density	5–15 mA/cm^2^
Na_2_WO_4_·2H_2_O	7.5 g/L-180 g/L	Duty cycle	10–30%
C_6_H_5_O_7_(NH_4_)_3_	100 g/L	PH	5
NaBr	5.4 g/L	Stirring rate	100 (r/min)
CH_4_N_2_S	2 drops	Temperature	30–50 °C
C_12_H_25_SO_4_Na	2 drops		

**Table 2:** Data processing.

m_x_/10^−2^ mg/cm^2^	3.763	3.235	2.861	3.437	3.179	3.521	3.433	2.974	3.472
V/(10^−2^ mg/cm^2^)^−1^	0.2657	0.3091	0.3495	0.2909	0.3146	0.2840	0.2913	0.3362	0.2880

**Table 3:** Levels and factors of the experiment.

Level	T/°C (T)	Current Density/mA/cm^2^ (C)	Duty Cycle (D)
1	30	5	0.1
2	40	10	0.2
3	50	15	0.3

**Table 4:** Results of the experiment.

Sample	T	C	D	V/(10^−2^ mg/cm^2^)^−1^
1	1 (30)	1 (5)	1 (0.1)	0.2657
2	1 (30)	2 (10)	2 (0.2)	0.3091
3	1 (30)	3 (15)	3 (0.3)	0.3495
4	2 (40)	1 (5)	1 (0.1)	0.2909
5	2 (40)	2 (10)	2 (0.2)	0.3146
6	2 (40)	3 (15)	3 (0.3)	0.2840
7	3 (50)	1 (5)	1 (0.1)	0.2913
8	3 (50)	2 (10)	2 (0.2)	0.3362
9	3 (50)	3 (15)	3 (0.3)	0.2880

**Table 5:** Comparison of experimental data with predicted values by BP ANN.

Sample	Experimental Value/%	Predicted Value/%	Relative Error/%
1	0.2657	0.2660	0.11
2	0.3091	0.3104	0.42
3	0.3495	0.3484	0.31
4	0.2909	0.2909	0
5	0.3146	0.3145	0.03
6	0.2840	0.2843	0.11
7	0.2913	0.2900	0.45
8	0.3362	0.3362	0
9	0.2880	0.2883	0.10

## Data Availability

The data presented in this study are available on request from the corresponding author.

## Appendix A

**Table A1:** Model Inputs and Symbols.

Symbols	Definition	Unit
Δ*M*	The oxidation weight gain of the coating	mg/cm^2^
*M*_1_	The mass of the sample before high-temperature oxidation	g
*M*_2_	The mass of the sample after high-temperature oxidation	g
*S*	The surface area of the sample	cm^2^
*m_x_*	The oxidative weight gain	g
*V*	The reciprocal of the oxidative weight gain	1/g

**Table A2:** Abbreviations in articles.

Symbols	Definition
BP	Backward propagation
ANN	Artificial neural network
Ni–W	Nickel and tungsten
HVOF	High velocity oxygen fuel
LY-12	A high strength duralumin
KSY	A muffle furnace model
SEM	Scanning electron microscope
EDX	Energy dispersive X-ray spectroscopy
EDS	Energy disperse spectroscopy
XRD	X-ray diffraction
VH	Vickers hardness
